# Basal and LPS-stimulated inflammatory markers and the course of individual symptoms of depression

**DOI:** 10.1038/s41398-020-00920-4

**Published:** 2020-07-15

**Authors:** Wessel A. van Eeden, Albert M. van Hemert, Ingrid V. E. Carlier, Brenda W. J. H. Penninx, Femke Lamers, Eiko I. Fried, Robert Schoevers, Erik J. Giltay

**Affiliations:** 1 Department of Psychiatry, Leiden University Medical Center, Leiden, The Netherlands; 2 Department of Psychiatry, Amsterdam Public Health Research Institute, Amsterdam UMC, Vrije Universiteit, Amsterdam, The Netherlands; 3 Department of Research and Innovation, GGZ inGeest, Amsterdam, The Netherlands; 4 Institute of Psychology, Leiden University, Leiden, The Netherlands; 5 Department of Psychiatry, University Medical Center Groningen, Groningen, The Netherlands

**Keywords:** Predictive markers, Depression, Diagnostic markers, Human behaviour

## Abstract

Multiple studies show an association between inflammatory markers and major depressive disorder (MDD). People with chronic low-grade inflammation may be at an increased risk of MDD, often in the form of sickness behaviors. We hypothesized that inflammation is predictive of the severity and the course of a subset of MDD symptoms, especially symptoms that overlap with sickness behavior, such as anhedonia, anorexia, low concentration, low energy, loss of libido, psychomotor slowness, irritability, and malaise. We tested the association between basal and lipopolysaccharide (LPS)-induced inflammatory markers with individual MDD symptoms (measured using the Inventory of Depressive Symptomatology Self-Report) over a period of up to 9 years using multivariate-adjusted mixed models in 1147–2872 Netherlands Study of Depression and Anxiety (NESDA) participants. At baseline, participants were on average 42.2 years old, 66.5% were women and 53.9% had a current mood or anxiety disorder. We found that basal and LPS-stimulated inflammatory markers were more strongly associated with sickness behavior symptoms at up to 9-year follow-up compared with non-sickness behavior symptoms of depression. However, we also found significant associations with some symptoms that are not typical of sickness behavior (e.g., sympathetic arousal among others). Inflammation was not related to depression as a unified syndrome but rather to the presence and the course of specific MDD symptoms, of which the majority were related to sickness behavior. Anti-inflammatory strategies should be tested in the subgroup of MDD patients who report depressive symptoms related to sickness behavior.

## Introduction

Inflammatory markers and depression have an intricate and complex relationship^1,2^. Evidence from meta-analyses suggests that depressed subjects have higher circulating concentrations of acute-phase proteins and pro-inflammatory cytokines compared with healthy subjects^3–8^. During an inflammatory response, the innate and adaptive immune systems are activated. Pro-inflammatory cytokines are produced by macrophages, monocytes, and other cells that stimulate the liver to produce acute-phase proteins. Chronically increased levels of peripheral blood interleukin (IL-6), tumor necrosis factor-alpha (TNF-α), and C-reactive protein (CRP), all of which indicate low-grade inflammation, are often associated with depression^1^. Other studies, however, have not found significant associations^7,8^.

Another approach to assess inflammation is to stimulating the immune cells and study the clinically important immune disturbances^9,10^. After ex vivo induction of lipopolysaccharide (LPS: the cell membrane of Gram-negative bacteria that strongly induces immunological responses) in whole blood samples, a wide array of pro-inflammatory cytokines are released, which can be measured in the supernatant^9,10^. Fewer studies exist on LPS-induced inflammation’s putative importance for depression^11–13^. Previous studies have found an association between LPS-stimulated inflammatory markers and depression. Sum scores of the Beck’s depression inventory were associated with higher levels of inflammatory markers interleukin-1beta (IL-1β), tumor necrosis factor-alpha (TNF-α), and interleukin-8 (IL-8), after LPS induction in whole blood. In addition, depressed men had higher monocyte chemoattractant protein-1 (MCP-1) levels, and depressed women had higher IL-1α levels^11,12^. In a previous cross-sectional analysis of the NESDA cohort, higher levels of LPS-induced inflammatory markers were found among patients with a remitted or current depression compared with healthy controls^13^. LPS-induced inflammatory markers were especially elevated among MDD patients with the DSM-5 anxious distress specifier^14^. Results remained statistically significant for LPS induced but not for basal levels of inflammatory markers, after adjusting for lifestyle and somatic health-related covariates^13^.

Researchers have speculated on the existence of crosstalk between several inflammatory pathways and neurocircuits that may lead to sickness behavior^1,15,16^. Sickness behavior as a syndrome is still rather ill-defined and has varied across time, disciplines, and studies but is generally regarded as an organized group of reward oriented behavioral and motivational changes that accompany inflammation and infections^1,15,17,18^. Researchers have theorized that sickness behavior holds some evolutionary advantages and has protective mechanisms for the individual (e.g., recovery), because it preserves energy resources needed for healing infection or other diseases and may help prevent the transmission of its potential infectious agent^1,18^. The causal chain may involve somatic triggers inducing an inflammatory response followed by sickness behavior. Sickness behavior in turn overlaps with and induces depression, with additional positive feedback loops between (neuro) inflammation and (neuro) degenerative processes^1,16,18^. Sickness behavior symptoms show a considerable overlap with depressive symptoms like anhedonia, anorexia, low concentration, low energy, low libido, psychomotor slowness, irritability; and researchers have hypothesized that depression is a maladaptive or exacerbated form of sickness behavior in some patients with chronic low-grade inflammation^15–20^. Besides their reward-sensitivity related symptoms, recent studies suggest that also trauma- and anxiety-related symptoms are related to inflammatory markers, resulting in a mix of overlapping symptoms of mood, anxiety, and post-traumatic stress disorder^13,21–23^. A causal pathway in which inflammation causes symptoms of anxiety is less established as studies show that inflammatory levels increase when study participants became anxious^24,25^, and a large longitudinal study found that anxiety predicted inflammation in the future but not vice versa^26^.

Inflammatory markers and depression have been linked, but effect sizes were generally small with limited clinical relevance for the individual patient^1^. Because depression is a heterogeneous disorder with large between-person variation^27^ and symptomatology^28,29^, low-grade inflammation may only be strongly linked to a subset of depressive symptoms^30,31^. Thus, inflammation may be involved in the pathogenesis of a subset of MDD patients. Identifying associations between pro-inflammatory markers and specific depressive symptoms could advance personalized medicine^32^. Nevertheless, few clinical studies have analyzed whether inflammatory markers are associated with specific MDD symptoms^30,32–34^.

Inflammation has been repeatedly linked to sickness-behavior symptoms such as certain sleeping problems, low energy, changes in appetite, low mood, and cognitive symptoms^30,32–34^. Two recent cross-sectional analyses in the current NESDA cohort found that inflammatory markers demonstrated the strongest associations with sleep and energy level, appetite/weight, and aches and pains, but associations were reduced or disappeared completely when adjusted for demographic-, lifestyle-, and disease-related factors such as BMI, activity, chronic somatic diseases, and gender^30,31^. Adjusting for certain variables is necessary in order to avoid confounding. However, overadjustment must also be avoided as variables such as activity, BMI, and somatic diseases may be part of the causal pathway between low-grade inflammation (which could be induced by somatic disease) on the one hand, and sickness behavior (which includes reduced activity and anorexia) and depression on the other hand^15,17–20^. There is still no consensus in the field about how to approach these demographic, somatic and lifestyle variables, and studies show that taking these variables into account as either confounders, or as part of the causal pathway, greatly influences the effect size of the relation between inflammation and depression^35^. We are not aware of previous studies that examined the symptom-specific associations with LPS-induced inflammatory agents. Moreover, examining individual symptoms longitudinally is important as inflammation may be related differently to depression symptoms longitudinally^36–40^. A recent longitudinal study for example found that inflammation was especially related to atypical symptoms^40^. Moreover, one meta-analysis demonstrated that increased inflammation can be associated with the development of late-life and the persistence of depression^39^. The present study extends on the current literature as we examined associations between basal levels and LPS-induced inflammatory markers and individual MDD symptoms in a large cohort over the course of 9 years. We hypothesized that persistent low-grade inflammation will show the strongest associations with symptoms characteristic of sickness behavior.

## Methods and materials

### Study sample and procedures

We evaluated baseline and follow-up data from 2872 out of 2981 participants from the NESDA cohort. NESDA investigated the course and consequences of depressive and anxiety disorders. NESDA included patients and healthy controls from a diverse array of (health-care) settings and applied a limited number of exclusion criteria, namely not being fluent in Dutch and the presence of other clinically overt psychiatric disorders (e.g., addiction, psychotic, and bipolar). With this method, NESDA aimed for a cohort that is representative for diverse populations of healthy controls and patients with depression and anxiety^41^. The first measurement wave (baseline) ran from 2004 to September 2007; the sixth wave at the 9-year follow-up finished in October 2016. All procedures involving human subjects/patients were approved by Ethical Review Board of the VU University Medical Centre and subsequently by local review boards of each participating center. Written informed consent was obtained from all participants. Where verbal consent was obtained this must be followed by a statement such as: Verbal consent was witnessed and formally recorded. More detailed design and sampling procedures are published elsewhere^41^. Basal serum levels of inflammatory markers were collected from 2867 participants. For logistical reasons, LPS induction in blood was only assessed during the last year of baseline sample collection. Consequently, data of LPS-stimulated inflammatory markers were available from 1229 out of 2867 participants. Of all the demographics and clinical characteristics mentioned in Table 1, this sub-selection did not differ from participants with missing data (*p* > 0.05), with the exception of age because the LPS subgroup was on average 1 year older. About 40% of the sample had a chronic somatic disease. A wide variety of diseases were assessed through a self-report questionnaire, asking for the presence of 20 common chronic diseases including asthma, chronic bronchitis or pulmonary emphysema, heart diseases or infarct, diabetes, stroke or CVA, arthritis or arthrosis, rheumatic complaints, tumor and/or metastasis, stomach or intestinal disorders, liver disease or liver cirrhosis, epilepsy, thyroid gland disease, or another chronic disease for which the patient receives treatment. A count was made of the number of chronic diseases for which a person reported receiving treatment. More details regarding this variable can be found elsewhere^42^.

### Measures

#### Demographics and clinical features

The Composite International Diagnostic Interview (CIDI WHO, version 2.1) was used to assess the presence of depressive and anxiety disorders according to the DSM-IV. The CIDI is a fully standardized diagnostic interview with validated psychometric characteristics^41,43^.

Demographic variables were described and included gender, age, ethnicity (yes/no regarding Northern European heritage), and level of education (elementary or less; general intermediate/secondary education; college/university). Patients also indicated whether they had a fever or cold in the week prior to blood draw (sickness prior to interview).

Medication use was determined by inspecting participants’ medication containers. Antidepressant use included selective serotonin reuptake inhibitors (SSRIs; ATC code: N06AB), tricyclic antidepressants (TCAs; ATC code: N06AA), and other antidepressants (ATC codes: N06AF, N06AG, N06AX). The use of statins (ATC code: C10AA) and anti-inflammatory, anti-rheumatic, and anti-allergic medications (ATC codes: M01A, M01B, A07EB, A07EC) was also assessed (further referred to as anti-inflammatory medication).

#### Independent variables: inflammatory markers

Baseline inflammatory markers CRP, IL-6, and TNF-α were assessed using fasting blood plasma levels (see the Supplementary information). Intra- and inter-assay coefficients of variation for CRP levels were 5% and 10%, respectively. Intra- and inter-assay coefficients of variation for IL-6 levels were 8% and 12%, respectively. Intra- and inter-assay coefficients of variation for TNF-α levels were 10% and 15%, respectively.

Inflammation is likely to occur when multiple cytokines are elevated. We did not form specific hypotheses about individual inflammatory markers, so we created a basal inflammation index, representing the mean value of log_e_-transformed (due to non-normality) and standardized levels of CRP, IL-6, and TNF-α^13^.

#### Independent variables: inflammatory markers after LPS induction

The innate immune response of 12 cytokines was assessed in ex vivo stimulated blood using LPS (see the Supplementary information). For all available samples, we simultaneously assessed levels of interferon-γ (IFN-γ), macrophage inflammatory protein-α (MIP-1α), IL-2, IL-6, IL-8, IL-10, IL-18, MCP-1, macrophage inflammatory protein-α (MIP-1α), MIP-1β, matrix metallopeptidase-2 (MMP-2), TNF-α, and TNF-β using a multi-analytic profile (Human CytokineMAP A v.1.0; Myriad RBM, Austin, TX, USA). Cytokine distributions were skewed to the right and therefore log_e_-transformed to normalize their distributions.

We created an LPS-induced inflammation index composed from the mean standardized value of all available LPS-induced markers, further referred to as LPS-induced inflammation index. To avoid loss of information, we conducted an exploratory factor analysis EFA^44^; which resulted into two LPS-induced inflammation indexes, further referred to as LPS-induced inflammation index-1 and LPS-induced inflammation index-2. Markers IFN-γ, IL-10, IL-2, IL-6, MMP-2, TNF-α, and TNF-β loaded on LPS-induced inflammation index-1 with factor loadings between 0.41 and 0.88 and a raw alpha of 0.86. IL-8, IL-18, MCP-1, MIP-1α, and MIP-1β loaded on LPS-induced inflammation index-2. See Supplementary information for the correlations between individual markers within each index (Supplementary information Fig. 1) and a more detailed description of the EFA procedures. Subsequently, two LPS-induced inflammation indexes were calculated as the mean of log_e_-transformed and standardized markers.

#### Dependent variables: IDS items

The sum score of the Inventory of Depressive Symptomatology Self-Report (IDS-SR) was used as the outcome measure for severity and course of depression on syndrome level, and the separate items were used for the symptom analyses^45,46^. The IDS-SR consists of 30 equally weighted items, rated on a 4-point Likert scale (0–3), and includes all symptoms of depression: melancholic, atypical, and anxious symptoms. Several additional symptoms were included: sympathetic arousal, pessimism, and interest in sex. We hypothesized that the following 16 IDS-SR items would be associated with inflammation at baseline because they can identify sickness-behavior symptoms:^15,17–20^ sleeping too much (Item 4), feeling irritable (Item 6), responsiveness of mood (Item 8), decrease in appetite (Item 11), decrease in weight (Item 12), concentration (Item 15), pessimism (Item 17), general interest (Item 19), low energy level (Item 20), capacity for pleasure (Item 21), interest in sex (Item 22), psychomotor retardation (Item 23), aches and pains (Item 25), sympathetic arousal (Item 26), constipation or diarrhea (Item 28), and leaden paralysis (Item 30).

### Statistical analysis

We used a multivariate linear mixed model with IDS-SR item-scores as outcome variables and inflammatory markers as the main independent variables. Because of the heterogeneity of our sample (healthy and depressed participants at baseline), the intercepts and slopes were considered as random variables, which resulted in a significantly better fit compared with a nonrandom model. (For the model with the basal inflammation index, the log likelihood (LL)-ratio increased by 80932.5, *p* < 0.001; for LPS-induced inflammation index-1, LL-ratio increased by 36887.2, *p* < 0.001; and for LPS-induced inflammation index-2, LL-ratio increased by 38640.1, *p* < 0.001.) Adding an interaction between time and inflammatory markers resulted a minimal increase of model fit. (For the model with the basal inflammation index, the LL-ratio increased by 12.2, *p* < 0.001; for LPS-induced inflammation index-1, the LL-ratio increased by 1.9, *p* = 0.167; and for LPS-induced inflammation index-2, the LL-ratio increased by 12.5, *p* < 0.001.) This small effect could be attributed to regression to the mean, so we decided not to include the interaction terms in our final models. Doing so resulted in mixed models for each individual IDS item with random intercepts and slopes over time, that analyzed whether participants with elevated levels of inflammation were more likely to have higher symptom levels at baseline and during the 9-year follow-up period. Models were adjusted for certain baseline variables: gender, age, sickness prior to interview, and the use of anti-inflammatory medication. In sensitivity analyses, we repeated the analysis for MDD patients (~30% of the total sample; Supplementary information Table 1 and Supplementary information Fig. 2) and for the LPS-inflammatory composite index score (Supplementary information Table 2 and Supplementary information Fig. 3). Moreover, sensitivity analyses were executed which additionally adjusted for chronic somatic diseases and antidepressants (Supplementary information Fig. 4). Subsequently, we adjusted the outcomes of the inflammation indexes for multiple testing using the Benjamin–Hochberg procedure^47^. Means of subscale scores (i.e., sickness behavior vs. non-sickness behavior) were computed and presented in line graphs for the effects over time. In order to yield beta coefficients that can be compared among symptoms, all outcome and independent variables were standardized (i.e., *z-*scores) with two-sided *p* values. All models were run in R, version 3.4.3.

## Results

### Sociodemographic and clinical characteristics at baseline

Our study sample was 66.7% female (*n* = 1975), and the ages ranged from 18 to 64 years at baseline (mean 42.9 years, SD 13.1; see Table 1 for demographics). The sample consisted of 35.4% 1-month recency MDD patients (*n* = 796), 2.8% with minor depression (*n* = 84), 9.3% with dysthymia (*n* = 277), 43.6% with a (comorbid) anxiety disorder (*n* = 1299), and 46.1% without a mood or anxiety diagnosis at baseline (*n* = 1368), of whom 54.2% never had a psychiatric diagnosis (*n* = 742).

**Table 1:** Sociodemographic and clinical characteristics.

	Whole sample	LPS-induced subsample
	*n* = 2872	*n* = 1229
Age in years (mean, SD)	41.9 (13.0)	42.8 (12.7)
Female (%)	66.5	65.6
North-European etnicity (%)	94.9	94.8
BMI (mean, SD)	25.6 (5.0)	25.7 (5.0)
Smoking status (%)		
Never smoker	28.0	29.0
Former smoker	33.6	34.2
Current smoker	38.4	36.8
Education level (%)		
Elementary or lower	6.49	6.4
Secondary education	58.2	56.7
College or university	35.4	36.9
Sickness prior to interview (%)	27.9	30.1
Chronic somatic disease, yes (%)	40.4	44.3
Anti-inflam. med., yes (%)	4.9	3.1
MDD, yes (%)	35.4	28.8
Minor depression, yes (%)	2.8	2.1
Dysthymia, yes (%)	9.3	10.4
Anxiety disorder, yes (%)	43.6	44.4
No Disorder (%)	46.1	46.3
No lifetime disorder (%)	34.1	36.3
Total score IDS at baseline (SD)	21.184 (14.6)	20.86 (14.6)
Antidepressants		
TCA (%)	3.7	2.9
SSRI (%)	16.8	16.5
Other (%)	5.5	5.6
No AD (%)	75.5	75.9
Inflammatory markers (mean, sd)		
TNF-α (pg/ml)	1.09 (1.41)	
IL-6 (pg/ml)	1.55 (13.5)	
CRP (mg/L)	2.82 (5.12)	
Inflammatory markers after LPS induction (mean, sd)		
IFN-ƴ (pg/ml)		12.80 (10.8)
IL-10 (pg/ml)		300.28 (294.9)
IL-18 (pg/ml)		262.39 (91.9)
IL-2 (pg/ml)		10.06 (5.0)
IL-6 (ng/ml)		27.36 (15.6)
IL-8 (ng/ml)		12.02 (7.7)
MCP-1 (ng/ml)		1.72 (1.1)
MIP-1α (ng/ml)		19.38 (12.0)
MIP-1β (ng/ml)		245.52 (123.3)
MMP-2 (pg/ml)		72.13 (19.3)
TNF-α (ng/ml)		3.19 (2.0)
TNF-β (pg/ml)		324.21 (126.6)

Demographic and clinical sample characteristics.

*BMI* body mass index, *MDD* major depressive disorder, *TCA* tricyclic antidepressants, *SSRI* selective serotonin reuptake inhibitors, *AD* antidepressants, *TNF* (median) tumor necrosis factor, *IL* Interleukin, *CRP* C-reactive protein, *IFN-ƴ* Interferon-ƴ, *MCP-1* higher monocyte chemoattractant protein-1, *MIP* macrophage inflammatory protein, *MMP-2* matrix metallopeptidase-2.

### Basal inflammation

We found a small but significant association between the basal inflammatory index and IDS-scores adjusted for age, gender, and anti-inflammatory medication (*β* = 0.039; *p* < 0.001). Thus, participants with a higher inflammatory index tended to have a 0.039 SD higher IDS-30 score over the course of 9 years, compared with participants with a 1 SD lower inflammatory index. This comes down to a absolute value of 1.12 IDS-SR sum score.

**Fig. 1:**
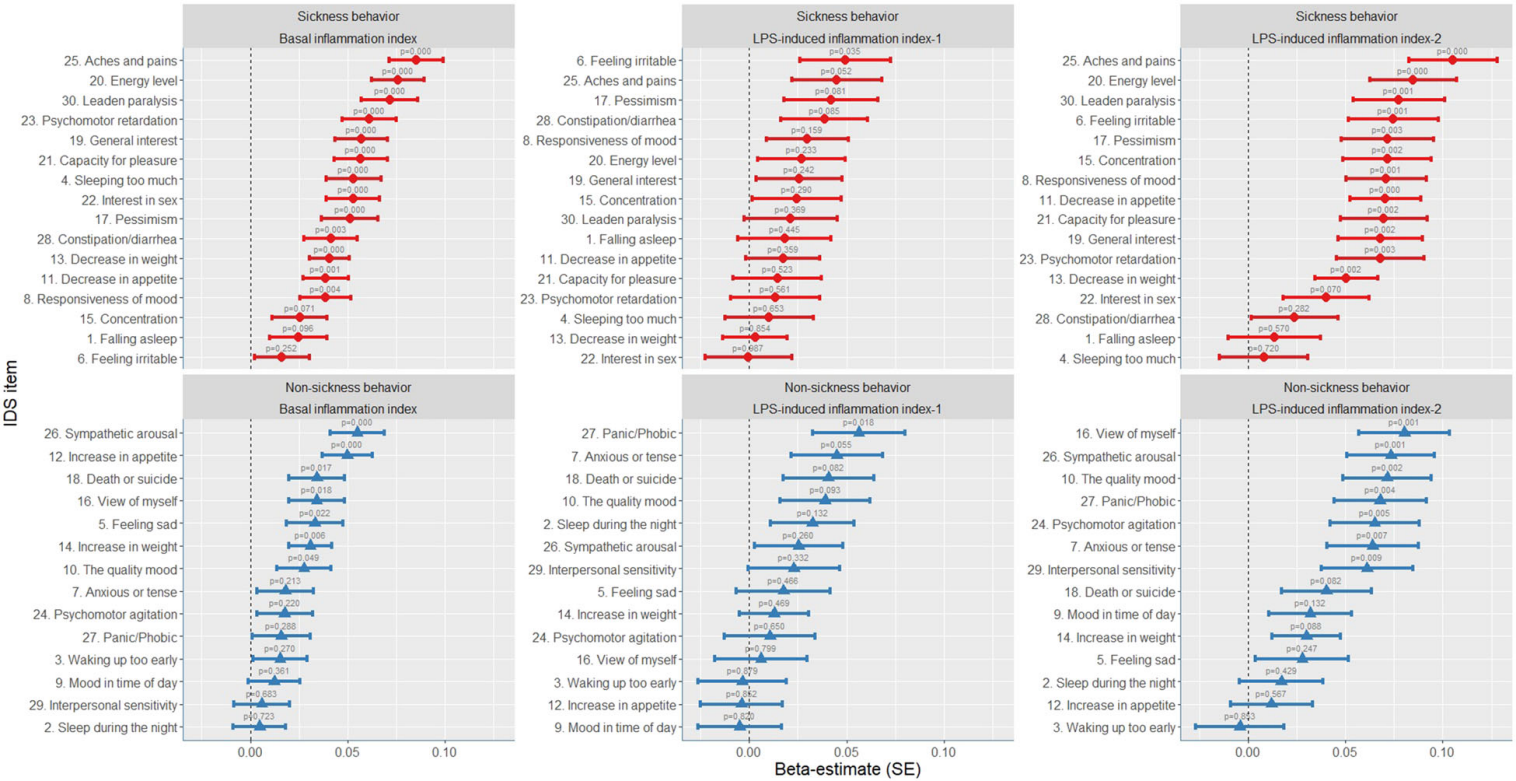
Associations of the basal inflammation index (*n* = 2872), LPS-induced inflammation index-1 (*n* = 1147), and LPS-induced inflammation index-2 (*n* = 1229) with individual depressive symptoms during 9 years. Standardized beta coefficients with error bars representing standard errors of the predictive values of inflammatory indexes in relation to individual depressive symptoms over 9 years of follow-up. The red dots represent depressive symptoms that are assumed to be related to sickness behavior. The blue dots represent depressive symptoms that are not related to sickness behavior. Beta coefficients translates a “the amount of SD that particular symptom is elevated averaged over 9 years, for each increased SD of inflammatory marker”. Assessments conducted using linear mixed models with repeated measures, adjusting for gender, age, use of anti-inflammatory drugs, and sickness prior to interview.

**Fig. 2:**
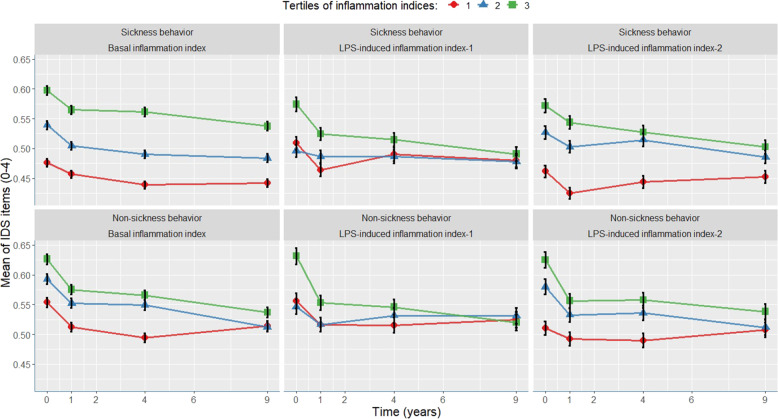
Tertiles of the basal inflammation index, LPS-induced inflammation index-1, and LPS-induced inflammation index-2 related to IDS-SR item-scores of sickness-behavior symptoms and non-sickness-behavior symptoms over the course of 9 years. Inflammation indexes are divided into tertiles of equal proportions of the sample distribution (1. lowest inflammatory markers: 0.0–0.33; 2. middle: 0.33–0.66; 3. highest: 0.66–1.0). Y-axis represent absolute mean values of IDS-SR item-scores (0–3). Error bars representing standard errors. IDS items related to sickness behavior: sleeping too much (Item 4), feeling irritable (Item 6), responsiveness of mood (Item 8), decrease in appetite (Item 11), decrease in weight (Item 12), concentration (Item 15), pessimism (Item 17), general interest (Item 19), low energy level (Item 20), capacity for pleasure (Item 21), interest in sex (Item 22), psychomotor retardation (Item 23), aches and pains (Item 25), constipation or diarrhea (Item 28) and leaden paralysis (Item 30). Non-sickness behavior IDS items: falling asleep (Item 1), sleep during the night (Item 2), waking up too early (Item 3), feeling sad (Item 5), anxious or tense (Item 7), mood in time of day (Item 9a), quality of mood (Item 10), increased appetite (Item 12), increased weight (Item 14), view of myself (Item 16), death or suicide (Item 18), psychomotor agitation (Item 24), sympathetic arousal (Item 26), panic/phobic (Item 27), and interpersonal sensitivity (Item 29).

Next, we analyzed the associations between the basal inflammation index for each of the 30 IDS items. Table 2 and Fig. 1 present the standardized beta coefficients of the basal inflammation index adjusted for age, gender, sickness prior to interview, and anti-inflammatory medication. All individual symptoms were positively related to high levels of basal inflammation. The beta sizes ranged from 0.005 (Item 2: Sleep during the night) to 0.085 (Item 25: Aches and pains). The course of tertiles of mean scores of sickness behavior symptoms vs. non-sickness behavior symptoms is presented in Fig. 2. As expected, both sub-scores declined steeply after baseline due to regression to the mean effects of anxiety and MDD patients who were initially selected for the NESDA cohort. Symptoms related to sickness behavior more strongly associated with basal inflammatory markers than other symptoms, the mean scores of which remained relatively elevated during the 9 years. Beta coefficients were statistically significant for quality of mood (Item 10; *β* = 0.028, *p* = 0.049) and all other items with beta coefficients above 0.028 (see Fig. 1). After adjusting for multiple testing for all tests summarized in Table 2, *p* values remained statistically significant for 17 items. Of the symptoms related to sickness behavior, 14 out of 16 symptoms were significantly associated with inflammation, compared with six out of 14 non-sickness-behavior symptoms. We found similar results with MDD patients only (*n* = 908), albeit with overall weaker effects due to lower variance and a smaller sample size (see Supplementary information Table 1 and Supplementary information Fig. 2). Among patients with MDD at baseline, eight out of 16 sickness-behavior-related symptoms were significantly associated with inflammation compared with three out of 14 non-sickness-behavior-related symptoms.

**Table 2:** (A) Basal serum inflammatory markers in realtion to IDS symptoms over the course of 9 years. (B) LPS-induced inflammatory markers in realtion to IDS symptoms over the course of 9 years.

A.	Basal Serum inflammation index		
	CRP, TNF-α, IL-6		
Item	Beta (SE)		*p* value
1. Falling asleep	0.025	(0.015)	0.096
2. Sleep during the night	0.005	(0.014)	0.723
3. Waking up too early	0.015	(0.014)	0.270
4. Sleeping too much	0.053	(0.014)	<0.001*
5. Feeling Sad	0.033	(0.014)	0.022*
6. Feeling irritable	0.016	(0.014)	0.252
7. Anxious or tense	0.018	(0.014)	0.213
8. Response of mood	0.038	(0.013)	0.004*
9a. Mood in time of day	0.012	(0.013)	0.361
10. Quality of mood	0.028	(0.014)	0.049*
11. Decreased appetite	0.039	(0.012)	0.001*
12. Increased appetite	0.050	(0.013)	<0.001*
13. Decreased weight	0.041	(0.010)	<0.001*
14. Increased weight	0.031	(0.011)	0.006*
15. Concentration	0.025	(0.014)	0.071
16. View of myself	0.034	(0.014)	0.018*
17. View of my future	0.051	(0.014)	<0.001*
18. Death or suicide	0.034	(0.014)	0.017*
19. General interest	0.057	(0.014)	<0.001*
20. Energy level	0.076	(0.014)	<0.001*
21. Capacity for pleasure	0.057	(0.014)	<0.001*
22. Interest in sex	0.053	(0.014)	<0.001*
23. Psychomotor retardation	0.061	(0.014)	<0.001*
24. Psychomotor agitation	0.018	(0.014)	0.220
25. Aches and pains	0.085	(0.014)	<0.001*
26. Sympathetic arousal	0.055	(0.014)	<0.001*
27. Panic/Phobic	0.016	(0.015)	0.288
28. Constipation/diarrhea	0.041	(0.014)	0.003*
29. Interpersonal sensitivity	0.006	(0.014)	0.683
30. Leaden paralysis	0.072	(0.014)	<0.001*

B.	LPS-induced index inflammation factor 1			LPS-induced index inflammation factor 2		
	IL-2, IL-6, IL-10, MMP-2, TNF-α, TNF-β, IFN-y			IL-8, IL-18, MCP-1, MIP-1α, MIP-1β		
Item	Beta (SE)		*p* value	Beta (SE)		*p* value
1. Falling asleep	0.018	(0.024)	0.445	0.013	(0.024)	0.570
2. Sleep during the night	0.032	(0.022)	0.132	0.017	(0.022)	0.429
3. Waking up too early	−0.003	(0.023)	0.879	−0.004	(0.023)	0.853
4. Sleeping too much	0.010	(0.023)	0.653	0.008	(0.023)	0.720
5. Feeling Sad	0.018	(0.024)	0.466	0.028	(0.024)	0.247
6. Feeling irritable	0.049	(0.023)	0.035*	0.075	(0.023)	0.001*
7. Anxious or tense	0.045	(0.023)	0.055	0.064	(0.023)	0.007*
8. Response of mood	0.030	(0.021)	0.159	0.071	(0.021)	0.001*
9a. Mood in time of day	−0.005	(0.021)	0.820	0.032	(0.021)	0.132
10. Quality of mood	0.039	(0.023)	0.093	0.071	(0.023)	0.002*
11. Decreased appetite	0.017	(0.019)	0.359	0.071	(0.018)	<0.001*
12. Increased appetite	−0.004	(0.021)	0.852	0.012	(0.021)	0.567
13. Decreased weight	0.003	(0.016)	0.854	0.050	(0.016)	0.002*
14. Increased weight	0.013	(0.018)	0.469	0.030	(0.018)	0.088
15. Concentration	0.024	(0.023)	0.290	0.071	(0.023)	0.002*
16. View of myself	0.006	(0.024)	0.799	0.080	(0.023)	0.001*
17. View of my future	0.042	(0.024)	0.081	0.072	(0.024)	0.003*
18. Death or suicide	0.041	(0.023)	0.082	0.040	(0.023)	0.082
19. General interest	0.026	(0.022)	0.242	0.068	(0.022)	0.002*
20. Energy level	0.027	(0.023)	0.233	0.085	(0.022)	<0.001*
21. Capacity for pleasure	0.014	(0.023)	0.523	0.070	(0.022)	0.002
22. Interest in sex	0.000	(0.022)	0.987	0.040	(0.022)	0.070
23. Psychomotor retardation	0.013	(0.023)	0.561	0.068	(0.022)	0.003*
24. Psychomotor agitation	0.011	(0.023)	0.650	0.065	(0.023)	0.005*
25. Aches and pains	0.045	(0.023)	0.052	0.105	(0.023)	<0.001*
26. Sympathetic arousal	0.025	(0.023)	0.260	0.073	(0.022)	0.001*
27. Panic/Phobic	0.056	(0.024)	0.018*	0.068	(0.024)	0.004*
28. Constipation/diarrhea	0.039	(0.022)	0.085	0.024	(0.022)	0.282
29. Interpersonal sensitivity	0.023	(0.024)	0.332	0.061	(0.023)	0.009*
30. Leaden paralysis	0.021	(0.024)	0.369	0.077	(0.023)	0.001*

Standardized beta coefficients of the association between basal serum inflammatory markers and individual depressive symptoms. Linear mixed models fitted with repeated measures, using standardized IDS-SR item-scores as outcome variables, which were assessed up to six times over 9 years of follow-up. Standardized beta coefficients were adjusted for gender, age, sickness prior to interview, and the use of anti-inflammatory medication.

*P values that remained significant (<0.05) after correcting for multiple testing using the Benjamin–Hochberg procedure.

### LPS-induced inflammation

The overall LPS-induced inflammation index (*β* = 0.036; *p* = 0.014) and the LPS-induced inflammation index-2 (*β* = 0.056, *p* < 0.001) were significantly related to the IDS score averaged over 30 items, and the LPS-induced inflammation index-1 indicated a relationship that approached significance (*β* = 0.026; *p* = 0.072). In absolute values this would translates in IDS-SR sum-scores difference of 1.12, 0.82, 1.71 for each SD increase of the LPS-induced inflammation index, LPS-induced inflammation index-1, and LPS-induced inflammation index-2, respectively.

The LPS-induced inflammation index-2 more strongly related to sickness-behavior symptoms, compared with non-sickness-behavior symptoms, than LPS-induced inflammation index-1, the beta coefficients of which ranged from −0.005 (mood related to time of the day) to 0.049 (feeling irritable) and were statistically significant for feeling irritable (Item 6; *β* = 0.049, *p* = 0.035) and panic/phobia (Item 27; *β* = 0.056, *p* = 0.018). After adjusting for multiple testing, only panic/phobia remained statistically significant.

Regarding LPS-induced inflammation index-2, beta coefficients ranged from −0.004 (waking up too early) to 0.105 (aches and pains). Betas were statistically significant for 13 out of 16 sickness-behavior symptoms and for six out of 14 non-sickness-behavior symptoms, with significant betas for decreased weight (Item 13; *β* = 0.050, *p* = 0.002) and all other items with betas >0.050 (see Fig. 1). Sickness-behavior symptoms remained elevated over the 9 years (Fig. 2). After adjusting for multiple testing, *p* values remained significant for 19 items.

In a sensitivity analysis, we analyzed the association of the composite LPS-induced inflammation index for all LPS-induced markers. Only seven out of 30 symptoms indicated significant associations (see Supplementary information Table 2 and Supplementary information Fig. 3). However, findings were no longer statistically significant after we adjusted for multiple testing. The LPS-induced inflammation index was equally related to sickness- and non-sickness-behavior symptoms.

## Discussion

We aimed to examine whether diverse inflammatory markers could predict the trajectories of individual symptoms of depression over the course of 9 years, specifically looking at symptoms indicative of sickness behavior. We found that the basal inflammation index and the LPS-induced inflammation index-2 predicted many depressive symptoms over the course of 9 years. By conducting regression analysis for each individual symptom separately, we demonstrated that significant associations between inflammatory markers and the course of a particular individual symptom was more than twice as likely to be significant when that symptom was related to sickness behavior compared with non-sickness-related behavior. The sickness-behavior theory may explain the rather weak (or sometimes conflicting) relationships found between low-grade inflammation and MDD^18^.

Four previous studies, three with cross-sectional^30,31,33^ and one with a prospective design^34^, have examined symptom-specific associations between basal serum inflammatory markers and depression. One study found that inflammation was specifically related to a change in appetite, poor sleep, and low energy^33^. Two of the cross-sectional studies were conducted within the current NESDA cohort and demonstrated that symptoms of sleeping problems, energy levels, appetite/weight changes, aches and pains and irritability were most likely to be positively associated with basal inflammatory markers^30,31^. By using network analyses, it was further demonstrated that the relation between basal inflammatory markers mostly runs through, and was affected by, lifestyle and disease-related covariates, such as BMI, activity level, and chronic somatic diseases^31^. Our study differed from these analyses because we used index scores instead of individual inflammatory markers. Moreover, as recommended for future research directions^31,48^, the individual symptoms were measured longitudinally at six time points over the course of 9 years. We adjusted for two disease-related variables (sickness prior to intake, and anti-inflammatory markers). Moreover, in a sensitivity analysis, we additionally adjusted for the count of self-report chronic somatic diseases and the use of antidepressants, which yielded a small attenuation of our results, but did not lead to different conclusions (Supplementary information Fig. 4). Our findings are largely consistent with previous findings; signs of low-grade inflammation at baseline were associated with the long-term symptomatology of sickness behavior^18^, and elevated levels of inflammation could lead to sickness behavior, which may explain some of the symptoms in certain cases of MDD^49–51^. However, we also found significant associations with symptoms that are not typical of sickness behavior (e.g., anxiety and low self-esteem). It is likely that much of the associations we found runs through lifestyle and disease-related variables, as these factors are thought to be part of the causal pathway^16,31,52^. It is hypothesized that (chronic) somatic factors results in higher levels of inflammatory markers, which in its turn results in sickness behavior (including lifestyle factors such as lower activity) which is related to, and is part of the depressive symptomatology^16,31,52^. Another line of thought is that these somatic and lifestyle factors act as confounding variables as they are both related to inflammation and depression^52^. The fact that we found the strongest association to symptoms that are specifically related to sickness behavior over the course of 9 years, suggests however that the sickness behavior theory is probable^16,53^.

To our knowledge, this is the first study that examined LPS-induced inflammatory markers in relation to the course of individual depressive symptoms. These markers reflect the cytokine production capacity when triggered by endogenous or exogenous triggers^9,54^, and are thought to be less affected by health and lifestyle factors such as BMI and chronic somatic diseases^13^. We found strong associations between LPS-induced inflammation index-2 markers and depressive symptoms. However, LPS-induced inflammation index-1 did not demonstrate such results. When looking at individual symptoms, LPS-induced, but not basal levels seem to be more specifically associated to symptoms of anxiety. Although this was not the focus of the current study, these findings are in line with the idea that anxiety-related symptoms may induce an inflammatory response^13,14,21^. Future research may focus on the potential role of LPS-induced markers in relation to the longitudinal course of anxiety-related symptoms.

Cytokines contribute to many aspects of human biology and have evolved to enable the sensing and interpretation of environmental cues relevant to maintaining a healthy physiology^55^. Although these secretory (glycol) proteins are best known for their role as custodians of immune homeostasis and the inflammatory response to infection, trauma, or injury, this study confirms their additional effects on mood and behavior^56^. Cytokines often display heterogenetic, pleiotropic, and overlapping functional properties^57^. Although cytokines are considered to be a “family,” this is a functional (rather than structural) concept. A common factor of the markers clustered in the LPS-induced inflammation index-2 is the link with T lymphocyte cells (T cells) and natural killer cells (NK cells). MCP-1, MIP-1α, and MIP-1β have a signaling function for monocytes and regulate T-cell activity. MIP-1β has an additional specificity for NK cells. IL-8 and IL-18 induce certain T-cell and NK-cell functions such as chemotaxis^58,59^ and locomotion^60,61^. There are indications that some MDD patients have impaired neuroprotective and anti-inflammatory T-cell responses^1^. Also, researchers have found a reduced number of circulating NK cells for MDD patients compared with healthy controls^1,62^.

Depression is a heterogeneous syndrome with a substantial variety of symptoms among patients with symptom-specific risk factors^63^. Not all patients exhibit symptoms related to sickness behavior, and only one-third of MDD patients exhibit elevated inflammatory markers^64^. Our findings could have implications for anti-inflammatory treatment^6,65^ and preventative care^66–69^ in a subgroup of depressed patients with sickness-behavior-related symptoms^70^. Research is underway to investigate the effects of anti-TNF-alpha biologic infliximab on measures of anhedonia, motivational behavior, and glutamatergic changes in the basal ganglia^71^ and to investigate the effects of simvastatin for treatment-resistant MDD^72^ and patients with comorbid obesity and MMD^73^. We recommend that future studies approach depression as a group of separate symptoms rather than as a unified construct. The construct of sickness behavior could be particularly promising in this regard.

Our study features several strengths, namely the substantial sample size and the 9-year follow-up period wherein we analyzed individual symptoms of depression. Multiple reviews have published about the sickness behavior theory and how this could relate to symptoms of depression. However, not many papers exist that tested how this theory translates to data of self-report symptoms of depression^1–5^. “This” study is novel in the sense that we explicitly categorized symptoms into sickness behavior symptoms and non-sickness behavior symptoms and found a convincing stronger association with the first. Moreover, a wide array of inflammatory markers were assessed at baseline, including LPS-induced markers. We did not have preliminary hypotheses regarding which markers would indicate certain depressive symptoms, so we constructed three inflammatory indexes based on inflammatory markers to enhance the interpretability of our results. We demonstrated the utility of these index scores for research purposes and it’s potential for clinical practice. By averaging multiple markers the effect of individual measurement errors is reduced, which is an important methodological advantage^74^. Some limitations must also be discussed. First, some of the component markers of the index scores were only weakly intercorrelated. Moreover, we composed two indexes based on data driven methods (Factor analysis^44^), more research is needed regarding grouping of individual markers based on underlying properties. Second, we repeatedly use the ill-defined term “sickness behavior”; different fields of medicine should solidify the definition so as to develop this construct in more depth^17,18^. Third, due to logistical reasons, LPS-stimulated markers were only assessed in a consecutive subsample of 1229 participants. Fourth, previous studies found that antidepressants might have anti-inflammatory effects. Rats treated with fluoxetine demonstrated lower IL-1β in plasma and brain after 90 and 120-day treatment^75^. Furthermore, two meta-analyses demonstrated that among MDD patients antidepressant treatment decreases TNF-α, IL-4, IL-6, IL-10 and IL-1ß^76,77^. In the present study, the use of antidepressants was not adjusted for in our first models, as their use may indicate more severe depressive symptoms (confounding-by-indication) and therefore may lead to overadjustment. However, sensitivity analyses demonstrate adding this variable as a confounder had only a limited effect on our outcomes and conclusions (Supplementary information Fig. 4). Finally, beta coefficients were statistically significant but still of small effect sizes, with questionable clinical relevance. However, self-reported IDS items were scored on crude four-point scales, potentially contributing to measurement error and reduced statistical power. Moreover, the NESDA cohort only used a single measurement of inflammatory markers; trajectory analyses with sequential day-to-day measures of inflammatory markers would have increased the precision of the independent variable.

In conclusion, we found that basal levels of inflammation and LPS-induced inflammatory markers predicted the course of individual depressive symptoms, especially those related to the construct of sickness behavior. This association persisted over the course of 9 years. Our findings suggest that inflammation might not relate to depression as one unified syndrome but rather to the presence and course of a subset of symptoms. Future studies should develop inflammation-targeted treatment strategies for individuals with symptom profiles associated with low-grade inflammation.

## Supplementary information

Supplementary Information

Supplementary Figure 1

Supplementary Figure 2

Supplementary Figure 3

Supplementary Figure 4

## References

[1] Miller AH, Raison CL (2016). The role of inflammation in depression: from evolutionary imperative to modern treatment target. Nat. Rev. Immunol..

[2] Krogh J (2014). The association between depressive symptoms, cognitive function, and inflammation in major depression. Brain, Behav. Immun..

[3] Dowlati Y (2010). A meta-analysis of cytokines in major depression. Biol. Psychiatry.

[4] Howren MB, Lamkin DM, Suls J (2009). Associations of depression with C-reactive protein, IL-1, and IL-6: a meta-analysis. Psychosom. Med..

[5] Liu Y, Ho RC-M, Mak A (2012). Interleukin (IL)-6, tumour necrosis factor alpha (TNF-α) and soluble interleukin-2 receptors (sIL-2R) are elevated in patients with major depressive disorder: a meta-analysis and meta-regression. J. Affect. Disord..

[6] Köhler O (2014). Effect of anti-inflammatory treatment on depression, depressive symptoms, and adverse effects: a systematic review and meta-analysis of randomized clinical trials. JAMA Psychiatry.

[7] Valkanova V, Ebmeier KP, Allan CL (2013). CRP, IL-6 and depression: a systematic review and meta-analysis of longitudinal studies. J. Affect. Disord..

[8] Haapakoski R, Mathieu J, Ebmeier KP, Alenius H, Kivimäki M (2015). Cumulative meta-analysis of interleukins 6 and 1β, tumour necrosis factor α and C-reactive protein in patients with major depressive disorder. Brain Behav. Immun..

[9] van der Linden MW, Huizinga TW, Stoeken DJ, Sturk A, Westendorp RG (1998). Determination of tumour necrosis factor-alpha and interleukin-10 production in a whole blood stimulation system: assessment of laboratory error and individual variation. J. Immunol. Methods.

[10] Kovach NL, Yee E, Munford RS, Raetz CR, Harlan JM (1990). Lipid IVA inhibits synthesis and release of tumor necrosis factor induced by lipopolysaccharide in human whole blood ex vivo. J. Exp. Med..

[11] Suarez EC, Krishnan RR, Lewis JG (2003). The relation of severity of depressive symptoms to monocyte-associated proinflammatory cytokines and chemokines in apparently healthy men. Psychosom. Med..

[12] Suarez EC, Lewis JG, Krishnan RR, Young KH (2004). Enhanced expression of cytokines and chemokines by blood monocytes to in vitro lipopolysaccharide stimulation are associated with hostility and severity of depressive symptoms in healthy women. Psychoneuroendocrinology.

[13] Vogelzangs N, De Jonge P, Smit J, Bahn S, Penninx B (2016). Cytokine production capacity in depression and anxiety. Transl. Psychiatry.

[14] Gaspersz R (2017). The role of anxious distress in immune dysregulation in patients with major depressive disorder. Transl. Psychiatry.

[15] Shattuck EC, Muehlenbein MP (2015). Human sickness behavior: ultimate and proximate explanations. Am. J. Phys. Anthropol..

[16] Maes M (2012). Depression and sickness behavior are Janus-faced responses to shared inflammatory pathways. BMC Med..

[17] Miller AH, Capuron L, Raison CL (2005). Immunologic influences on emotion regulation. Clin. Neurosci. Res..

[18] Dantzer R, Kelley KW (2007). Twenty years of research on cytokine-induced sickness behavior. Brain Behav. Immun..

[19] Vollmer-Conna U (2004). Production of pro-inflammatory cytokines correlates with the symptoms of acute sickness behaviour in humans. Psychological Med..

[20] Smith AP (2012). Effects of the common cold on mood, psychomotor performance, the encoding of new information, speed of working memory and semantic processing. Brain Behav. Immun..

[21] Felger JC (2018). Imaging the role of inflammation in mood and anxiety-related disorders. Curr. Neuropharmacol..

[22] Naude PJW, Roest AM, Stein DJ, de Jonge P, Doornbos B (2018). Anxiety disorders and CRP in a population cohort study with 54,326 participants: the LifeLines study. World J. Biol. Psychiatry.

[23] Renna ME, O’Toole MS, Spaeth PE, Lekander M, Mennin DS (2018). The association between anxiety, traumatic stress, and obsessive–compulsive disorders and chronic inflammation: a systematic review and meta‐analysis. Depress Anxiety.

[24] Bierhaus A (2003). A mechanism converting psychosocial stress into mononuclear cell activation. Proc. Natl Acad. Sci..

[25] Pace TW (2006). Increased stress-induced inflammatory responses in male patients with major depression and increased early life stress. Am. J. Psychiatry.

[26] Glaus J (2018). The bidirectional relationship between anxiety disorders and circulating levels of inflammatory markers: results from a large longitudinal population‐based study. Depress Anxiety.

[27] Sjoholm L, Lavebratt C, Forsell Y (2009). A multifactorial developmental model for the etiology of major depression in a population-based sample. J. Affect Disord..

[28] Fried Nesse (2015). Depression is not a consistent syndrome: an investigation of unique symptom patterns in the STAR* D study. J. Affect. Disord..

[29] van Eeden WA, van Hemert AM, Carlier IVE, Penninx BW, Giltay EJ (2019). Severity, course trajectory, and within-person variability of individual symptoms in patients with major depressive disorder. Acta Psychiatr. Scand..

[30] Lamers, F., Milaneschi, Y., De Jonge, P., Giltay, E. J. & Penninx, B. W. J. H. Metabolic and inflammatory markers: associations with individual depressive symptoms. *Psychol. Med.* **48**, 1102–1110 (2018). 10.1017/S0033291717002483 28889804

[31] Fried, E., Von Stockert, S., Haslbeck, J., Lamers, F., Schoevers, R. & Penninx, B. Using network analysis to examine links between individual depression symptoms, inflammatory markers, and covariates. *Psychol. Med*. 1–9, https://www.cambridge.org/core/journals/psychologicalmedicine/article/using-network-analysis-to-examine-links-between-individual-depressive-symptoms-inflammatory-markers-andcovariates/E2C8D6857450A832AF10CD9E8DA757BB (2019). 10.1017/S0033291719002770 31615595

[32] Köhler-Forsberg O (2017). Association between C-reactive protein (CRP) with depression symptom severity and specific depressive symptoms in major depression. Brain Behav. Immun..

[33] Jokela M, Virtanen M, Batty GD, Kivimäki M (2016). Inflammation and specific symptoms of depression. JAMA Psychiatry.

[34] White J, Kivimäki M, Jokela M, Batty GD (2017). Association of inflammation with specific symptoms of depression in a general population of older people: the English Longitudinal Study of Ageing. Brain Behav. Immun..

[35] Horn SR (2018). Replication and reproducibility issues in the relationship between C-reactive protein and depression: a systematic review and focused meta-analysis. Brain Behav. Immun..

[36] Uher R (2014). An inflammatory biomarker as a differential predictor of outcome of depression treatment with escitalopram and nortriptyline. Am. J. Psychiatry.

[37] Huang M (2019). Longitudinal association of inflammation with depressive symptoms: a 7-year cross-lagged twin difference study. Brain Behav. Immun..

[38] Lamers, F., Milaneschi, Y., Smit, J. H., Schoevers, R. A., Wittenberg, G. & Penninx, B. W. Longitudinal association between depression and inflammatory markers: results from the Netherlands Study of depression and anxiety. *Biol. Psychiatry* **85**, 829–837. https://www.cambridge.org/core/journals/psychologicalmedicine/article/using-network-analysis-to-examine-links-between-individual-depressive-symptoms-inflammatory-markers-andcovariates/E2C8D6857450A832AF10CD9E8DA757BB (2019). 10.1016/j.biopsych.2018.12.020 30819515

[39] Ng A (2018). IL-1beta, IL-6, TNF- alpha and CRP in Elderly Patients with Depression or Alzheimer’s disease: systematic review and meta-analysis. Sci. Rep..

[40] Lamers, F., Milaneschi, Y., Vinkers, C. H., Schoevers, R. A., Giltay, E. J. & Penninx, B. W. Depression profilers and immuno-metabolic dysregulation: longitudinal results from the NESDA study. *Brain Behav. Immun*. https://www.sciencedirect.com/science/article/pii/S0889159120301203?casa_token=mwwdWhSwtqsAAAAA:M2jH-2ngI_wgw_YaeEedUDSNhTKU5AJcWy_USL3r7bqD09jIPD2Z8xVq2iEnj2vVOTK_gxgjeQ (2020). 10.1016/j.bbi.2020.04.002 32272220

[41] Penninx BW (2008). The Netherlands Study of Depression and Anxiety (NESDA): rationale, objectives and methods. Int J. Methods Psychiatr. Res..

[42] Gerrits MM, van Oppen P, van Marwijk HW, van der Horst H, Penninx BW (2013). The impact of chronic somatic diseases on the course of depressive and anxiety disorders. Psychother. Psychosom..

[43] Wittchen H-U (1994). Reliability and validity studies of the WHO-Composite International Diagnostic Interview (CIDI): a critical review. J. Psychiatr. Res..

[44] Bandalos, D. L., Finney, S. J. Factor analysis: Exploratory and confirmatory. *The reviewer’s guide to quantitative methods in the social sciences*. 98–122 (Routledge, 2018).

[45] Trivedi MH (2004). The Inventory of Depressive Symptomatology, Clinician Rating (IDS-C) and Self-Report (IDS-SR), and the Quick Inventory of Depressive Symptomatology, Clinician Rating (QIDS-C) and Self-Report (QIDS-SR) in public sector patients with mood disorders: a psychometric evaluation. Psychol. Med..

[46] Rush AJ, Gullion CM, Basco MR, Jarrett RB, Trivedi MH (1996). The Inventory of Depressive Symptomatology (IDS): psychometric properties. Psychol. Med..

[47] Benjamini, Y., Hochberg, Y. Controlling the false discovery rate: a practical and powerful approach to multiple testing. *J. Royal Stat. Soc. Series B (Methodological)* **57**, 289–300 (1995).

[48] Smith KJ, Au B, Ollis L, Schmitz N (2018). The association between C-reactive protein, Interleukin-6 and depression among older adults in the community: a systematic review and meta-analysis. Exp. Gerontol..

[49] Haroon, E. et al. Antidepressant treatment resistance is associated with increased inflammatory markers in patients with major depressive disorder. *Psychoneuroendocrinology* **95**, 43–49 (2018). 10.1016/j.psyneuen.2018.05.026 PMC6427066 29800779

[50] Duivis HE, Vogelzangs N, Kupper N, de Jonge P, Penninx BW (2013). Differential association of somatic and cognitive symptoms of depression and anxiety with inflammation: findings from the Netherlands Study of Depression and Anxiety (NESDA). Psychoneuroendocrinology.

[51] Smith RS (1991). The macrophage theory of depression. Med. Hypotheses.

[52] Berk M (2013). So depression is an inflammatory disease, but where does the inflammation come from?. BMC Med..

[53] Rosenblat JD, Cha DS, Mansur RB, Mcintyre RS (2014). Inflamed moods: a review of the interactions between inflammation and mood disorders. Prog. Neuro-Psychopharmacol. Biol. Psychiatry.

[54] van den Biggelaar AH (2007). Inflammation and interleukin-1 signaling network contribute to depressive symptoms but not cognitive decline in old age. Exp. Gerontol..

[55] Walker KJ (2011). Chemokines: Types, Functions, and Structural Characteristics..

[56] Dinarello CA (2007). Historical insights into cytokines. Eur. J. Immunol..

[57] Jones, S. A., Jenkins, B. J. Recent insights into targeting the IL-6 cytokine family in inflammatory diseases and cancer. *Nature Rev. Immunol.* **18**, 773–789 (2018). 10.1038/s41577-018-0066-7 30254251

[58] Srivastava S (2013). Effects of interleukin-18 on natural killer cells: costimulation of activation through Fc receptors for immunoglobulin. Cancer Immunol. Immunother..

[59] Wechsler AS, Gordon MC, Dendorfer U, LeClair KP (1994). Induction of IL-8 expression in T cells uses the CD28 costimulatory pathway. J. Immunol..

[60] Sebok K (1993). IL-8 induces the locomotion of human IL-2-activated natural killer cells. Involvement of a guanine nucleotide binding (Go) protein. J. Immunol..

[61] Komai-Koma M (2003). Chemoattraction of human T cells by IL-18. J. Immunol..

[62] Majidi-Zolbanin, S. et al. Correlation between major depressive disorder and circulating natural killer cells. *Arch. Med. Lab. Sci.* **1**, http://journals.sbmu.ac.ir/archives/article/view/10245 (2019).

[63] Fried Nesse, Zivin Guille, Sen (2014). Depression is more than the sum score of its parts: individual DSM symptoms have different risk factors. Psychol. Med..

[64] Raison CL, Miller AH (2011). Is depression an inflammatory disorder?. Curr. Psychiatry Rep..

[65] Jha M, Trivedi M (2018). Personalized antidepressant selection and pathway to novel treatments: clinical utility of targeting inflammation. Int. J. Mol. Sci..

[66] Gimeno D (2009). Associations of C-reactive protein and interleukin-6 with cognitive symptoms of depression: 12-year follow-up of the Whitehall II study. Psychological Med..

[67] Au B, Smith KJ, Gariépy G, Schmitz N (2015). The longitudinal associations between C‐reactive protein and depressive symptoms: evidence from the English Longitudinal Study of Ageing (ELSA). Int. J. Geriatr. Psychiatry.

[68] Ridker PM, Buring JE, Shih J, Matias M, Hennekens CH (1998). Prospective study of C-reactive protein and the risk of future cardiovascular events among apparently healthy women. Circulation.

[69] Ridker PM, Hennekens CH, Buring JE, Rifai N (2000). C-reactive protein and other markers of inflammation in the prediction of cardiovascular disease in women. N. Engl. J. Med..

[70] Kopschina Feltes P (2017). Anti-inflammatory treatment for major depressive disorder: implications for patients with an elevated immune profile and non-responders to standard antidepressant therapy. J. Psychopharmacol..

[71] Lee Y (2018). Anti-cytokine agents for anhedonia: targeting inflammation and the immune system to treat dimensional disturbances in depression. Ther. Adv. Psychopharmacol..

[72] Husain, M. I. et al. Adjunctive simvastatin for treatment-resistant depression: study protocol of a 12-week randomised controlled trial. *BJPsych Open* **5**, e13 (2019). 10.1192/bjo.2018.84 PMC6381416 30762508

[73] Otte, C. Simvastatin add-on to Escitalopram in patients with comorbid obesity and major depression: a multicenter, randomized, double-blind, placebo-controlled trial; EudraCT number 2018-002947-27. (Charité – Universitätsmedizin Berlin: Germany, 2019). 10.1136/bmjopen-2020-040119 PMC7709515 33262189

[74] Hardikar S (2014). Intraindividual variability over time in plasma biomarkers of inflammation and effects of long-term storage. Cancer Causes Control.

[75] Lu Y (2017). Chronic administration of fluoxetine and pro-inflammatory cytokine change in a rat model of depression. PLoS ONE.

[76] Wiedlocha M (2018). Effect of antidepressant treatment on peripheral inflammation markers - a meta-analysis. Prog. Neuropsychopharmacol. Biol. Psychiatry.

[77] Hannestad J, DellaGioia N, Bloch M (2011). The effect of antidepressant medication treatment on serum levels of inflammatory cytokines: a meta-analysis. Neuropsychopharmacology.

