# Supplementary material for: Basal and LPS-stimulated inflammatory markers and the course of individual symptoms of depression
Source: Transl Psychiatry. 2020 Jul 15;10:235. doi: 10.1038/s41398-020-00920-4 (PMC7363825; doi:10.1038/s41398-020-00920-4)
Supplement: Supplementary file 1 — Supplementary Information [file 41398_2020_920_MOESM1_ESM.docx]

# Supplementary Information

### **Basal inflammation data collection procedure**

Baseline inflammatory markers CRP, IL-6, and TNF-α were determined from fasting blood plasma. After an overnight fast, 50 ml of blood was drawn, immediately transferred to a local laboratory, and kept frozen at $-$80°C. High sensitivity plasma levels of CRP were measured in duplicate by an in-house, high sensitivity enzyme-linked immunosorbent assay (ELISA), which is based on a purified protein and polyclonal anti-CRP antibodies (Dako, Glostrup, Denmark). The lower detection limit of CRP is 0.1 mg/l, and the sensitivity is 0.05 mg/l. Intra- and inter-assay coefficients of variation were 5% and 10%, respectively. Plasma IL-6 levels were measured in duplicate by a high sensitivity ELISA (PeliKine Compact™ ELISA, Sanquin, Amsterdam, the Netherlands). The lower detection limit of IL-6 is 0.35 pg/ml, and the sensitivity is 0.10 pg/ml. Intra- and inter-assay coefficients of variation were 8% and 12%, respectively. Plasma TNF-α levels were assayed in duplicate using a high sensitivity solid phase ELISA (Quantikine HS Human TNF-α Immunoassay, R&D systems, Minneapolis, MN, USA). The lower detection limit of TNF-α is 0.10 pg/ml, and the sensitivity is 0.11 pg/ml. Intra- and inter-assay coefficients of variation were 10% and 15%, respectively.

### **Inflammatory markers after the LPS-induction data-collection procedure**

The innate immune response of 12 cytokines was assessed in blood that was ex vivo stimulated with LPS. Venous whole blood samples were obtained at baseline in single 7-ml heparin-coated tubes (Greiner Bio-One, Monroe, NC, USA). Between 10 and 60 min after blood draw, 2.5 ml of blood was transferred into a PAXgene tube (Qiagen, Valencia, CA, USA). Remaining blood (4.5 ml) was stimulated by addition of LPS (10 ng/ml − 1 blood; Escherichia coli, Sigma, St. Louis, MO, USA). LPS-stimulated samples were laid flat and incubated at a slow rotation for 5–6 hr at 37°C. A 2.5 ml sample of this LPS-stimulated blood was transferred into a PAXgene tube. This LPS procedure was carried out at four laboratories (Amsterdam, Leiden, Groningen, and Heerenveen, The Netherlands).

Levels of interferon-γ (IFN-γ), macrophage inflammatory protein-α (MIP-1α), IL-2, IL-6, IL-8, IL-10, IL-18, MCP-1, macrophage inflammatory protein-α (MIP-1α), MIP-1β, matrix metallopeptidase-2 (MMP-2), TNF-α, and TNF-β were assessed simultaneously for all available samples, using a multi-analytic profile (Human CytokineMAP A v.1.0; Myriad RBM, Austin, TX, USA). This commercial platform adheres to stringent guidelines of quality control and has Clinical Laboratory Improvement Amendments (CLIA) approval. Cytokine distributions were skewed to the right and therefore log_e_-transformed to normalize their distributions.

We created an LPS-induced inflammation index from the mean standardized value of all available LPS-induced markers, further referred to as the LPS-induced inflammation index. To avoid loss of information, we conducted an exploratory factor analysis (EFA) with promax rotation on all LPS-induced inflammatory markers in order to reduce these into additional data-driven index scores. This resulted into two LPS-induced inflammation indexes with an eigenvalue of 0.55, further referred to as LPS-induced inflammation index-1 and LPS-induced inflammation index-2. Markers IFN-γ, IL-10, IL-2, IL-6, MMP-2, TNF-α, and TNF-β loaded on LPS-induced inflammation index-1 with factor loadings between 0.41 and 0.88 and a raw alpha of 0.86. IL-8, IL-18, MCP-1, MIP-1α, and MIP-1β loaded on LPS-induced inflammation index-2 with loadings between 0.34 and 0.94 and a raw alpha of 0.89. This two-factor solution fitted the data better but still poorly—Comparative Fit Index (CFI) = 0.867, Tucker-Lewis index (TLI) = 0.796, Root Mean Square Error of Approximation (RMSEA) = 0.187—compared to a one-factor solution: CFI = 0.794, TLI = 0.748, and RMSEA = 0.208. See SI Figure 1 for the correlations between individual markers within each index. Subsequently, two LPS-induced inflammation indexes were calculated as the mean of log_e_-transformed and standardized markers.

| **SI Table 1. IDS symptoms over the course of nine years in relation to inflammatory markers for MDD patients only** | | | | | | | | | |
| --- | --- | --- | --- | --- | --- | --- | --- | --- | --- |
|  | Basal Serum inflammation index | | | LPS-induced index inflammation factor 1 | | | LPS-induced index inflammation factor 2 | | |
|  | CRP, TNF-α, IL-6 | | | IL-2, IL-6, IL-10, MMP-2, TNF-α, TNF-β, IFN-y | | | IL-8, IL-18, MCP-1, MIP-1α, MIP-1β | | |
| **Item** | Beta (SE) | | p-value | Beta (SE) | | p-value | Beta (SE) | | p-value |
| 1. Falling asleep | -0.001 | (0.026) | 0.955 | -0.055 | (0.046) | 0.229 | -0.026 | (0.049) | 0.598 |
| 2. Sleep during the night | 0.023 | (0.022) | 0.286 | -0.013 | (0.037) | 0.723 | 0.015 | (0.041) | 0.709 |
| 3. Waking up too early | 0.043 | (0.025) | 0.087 | -0.054 | (0.045) | 0.231 | -0.026 | (0.048) | 0.584 |
| 4. Sleeping too much | 0.016 | (0.026) | 0.544 | -0.022 | (0.044) | 0.623 | 0.003 | (0.048) | 0.946 |
| 5. Feeling Sad | 0.045 | (0.021) | 0.034* | 0.002 | (0.038) | 0.951 | 0.067 | (0.041) | 0.104 |
| 6. Feeling irritable | 0.008 | (0.021) | 0.712 | 0.021 | (0.038) | 0.572 | 0.101 | (0.040) | 0.013* |
| 7. Anxious or tense | 0.004 | (0.022) | 0.843 | 0.002 | (0.038) | 0.965 | 0.086 | (0.041) | 0.040 |
| 8. Response of mood | 0.027 | (0.025) | 0.273 | -0.027 | (0.043) | 0.532 | 0.017 | (0.045) | 0.703 |
| 9a. Mood in time of day | -0.036 | (0.025) | 0.153 | 0.020 | (0.044) | 0.649 | 0.029 | (0.048) | 0.536 |
| 10. Quality of mood | 0.047 | (0.023) | 0.038 | 0.008 | (0.040) | 0.840 | 0.081 | (0.043) | 0.059 |
| 11. Decreased appetite | 0.035 | (0.023) | 0.140 | 0.008 | (0.040) | 0.844 | 0.091 | (0.042) | 0.029* |
| 12. Increased appetite | 0.077 | (0.026) | 0.003* | -0.022 | (0.046) | 0.641 | -0.024 | (0.050) | 0.633 |
| 13. Decreased weight | 0.018 | (0.019) | 0.492 | -0.028 | (0.033) | 0.409 | 0.051 | (0.035) | 0.139 |
| 14. Increased weight | 0.031 | (0.020) | 0.120 | 0.011 | (0.035) | 0.752 | 0.039 | (0.038) | 0.298 |
| 15. Concentration | 0.018 | (0.021) | 0.378 | 0.030 | (0.036) | 0.409 | 0.092 | (0.039) | 0.019* |
| 16. View of myself | 0.043 | (0.024) | 0.078 | 0.023 | (0.043) | 0.585 | 0.114 | (0.046) | 0.014* |
| 17. View of my future | 0.045 | (0.020) | 0.024* | 0.041 | (0.034) | 0.228 | 0.093 | (0.037) | 0.014* |
| 18. Death or suicide | 0.041 | (0.027) | 0.130 | 0.007 | (0.047) | 0.890 | 0.032 | (0.050) | 0.532 |
| 19. General interest | 0.055 | (0.024) | 0.020* | -0.032 | (0.042) | 0.453 | 0.051 | (0.045) | 0.255 |
| 20. Energy level | 0.059 | (0.021) | 0.004* | -0.030 | (0.036) | 0.410 | 0.015 | (0.039) | 0.699 |
| 21. Capacity for pleasure | 0.056 | (0.024) | 0.020* | -0.030 | (0.043) | 0.474 | 0.042 | (0.046) | 0.357 |
| 22. Interest in sex | 0.037 | (0.023) | 0.112 | -0.003 | (0.043) | 0.948 | -0.008 | (0.046) | 0.860 |
| 23. Psychomotor retardation | 0.067 | (0.027) | 0.013* | -0.042 | (0.048) | 0.380 | 0.058 | (0.051) | 0.258 |
| 24. Psychomotor agitation | 0.016 | (0.025) | 0.518 | 0.020 | (0.044) | 0.651 | 0.070 | (0.047) | 0.136 |
| 25. Aches and pains | 0.096 | (0.021) | <0.001* | 0.014 | (0.038) | 0.703 | 0.122 | (0.041) | 0.003* |
| 26. Sympathetic arousal | 0.048 | (0.022) | 0.030* | 0.001 | (0.040) | 0.973 | 0.095 | (0.043) | 0.029* |
| 27. Panic/Phobic | -0.008 | (0.025) | 0.758 | -0.002 | (0.045) | 0.969 | 0.017 | (0.048) | 0.721 |
| 28. Constipation/diarrhea | 0.031 | (0.024) | 0.201 | -0.009 | (0.043) | 0.832 | 0.007 | (0.046) | 0.879 |
| 29. Interpersonal sensitivity | -0.022 | (0.022) | 0.325 | 0.003 | (0.038) | 0.940 | 0.106 | (0.042) | 0.011* |
| 30. Leaden paralysis | 0.043 | (0.020) | 0.028* | -0.006 | (0.034) | 0.861 | 0.015 | (0.037) | 0.687 |

**SI Table 1. Standardized beta coefficients of the association between inflammatory markers and individual depressive symptoms in a sample of MDD patients only.** Standardized beta coefficients of linear mixed models with basal inflammation index and LPS-induced inflammation index-1 and -2 assessed with repeated measures, used to predict standardized IDS-SR item-scores measured over 9 years of follow up. Assessed at up to six time-points, adjusted for baseline variables of gender, age, sickness prior to interview, and the use of anti-inflammatory medication in a sample of MDD patients only.
**P* values that remained significant (< 0.05) after correcting for multiple testing using the Benjamin–Hochberg procedure.

| **SI Table 2. IDS symptoms over the course of nine years in relation to LPS-induced inflammatory markers** | | | |
| --- | --- | --- | --- |
|  | LPS-induced Inflammationindex | | |
|  | IL-2, IL-6, IL-10, MMP-2, TNF-α, TNF-β, IFN-y, IL-8, IL-18, MCP-1, MIP-1α, MIP-1β | | |
| **Item** | Beta (SE) | | p-value |
| 1. Falling asleep | 0.014 | (0.024) | 0.552 |
| 2. Sleep during the night | 0.029 | (0.022) | 0.186 |
| 3. Waking up too early | 0.000 | (0.023) | 0.989 |
| 4. Sleeping too much | 0.013 | (0.023) | 0.569 |
| 5. Feeling Sad | 0.025 | (0.024) | 0.315 |
| 6. Feeling irritable | 0.060 | (0.024) | 0.011* |
| 7. Anxious or tense | 0.051 | (0.024) | 0.033* |
| 8. Response of mood | 0.041 | (0.021) | 0.058 |
| 9a. Mood in time of day | 0.003 | (0.022) | 0.894 |
| 10. Quality of mood | 0.047 | (0.023) | 0.045 |
| 11. Decreased appetite | 0.035 | (0.019) | 0.064 |
| 12. Increased appetite | 0.000 | (0.021) | 0.993 |
| 13. Decreased weight | 0.021 | (0.017) | 0.198 |
| 14. Increased weight | 0.017 | (0.018) | 0.342 |
| 15. Concentration | 0.041 | (0.023) | 0.082 |
| 16. View of myself | 0.030 | (0.024) | 0.208 |
| 17. View of my future | 0.055 | (0.024) | 0.023* |
| 18. Death or suicide | 0.039 | (0.024) | 0.098 |
| 19. General interest | 0.037 | (0.022) | 0.094 |
| 20. Energy level | 0.044 | (0.023) | 0.052 |
| 21. Capacity for pleasure | 0.030 | (0.023) | 0.195 |
| 22. Interest in sex | 0.012 | (0.023) | 0.586 |
| 23. Psychomotor retardation | 0.032 | (0.023) | 0.163 |
| 24. Psychomotor agitation | 0.021 | (0.024) | 0.381 |
| 25. Aches and pains | 0.066 | (0.023) | 0.005* |
| 26. Sympathetic arousal | 0.051 | (0.023) | 0.026* |
| 27. Panic/Phobic | 0.062 | (0.024) | 0.010* |
| 28. Constipation/diarrhea | 0.040 | (0.023) | 0.080 |
| 29. Interpersonal sensitivity | 0.035 | (0.024) | 0.141 |
| 30. Leaden paralysis | 0.045 | (0.024) | 0.064 |

**SI Table 2: Standardized beta coefficients of the association between LPS-induced inflammatory markers and individual depressive symptoms.** Standardized beta coefficients of the LPS-induced inflammation index, assessed using a mixed model with repeated measures with standardized IDS-SR item score as the outcome variable. Assessed at six time points over the 9 years of follow up and adjusted for baseline variables of gender, age, sickness prior to interview, and the use of anti-inflammatory medication.
**P* values that remained significant (< 0.05) after correcting for multiple testing using the Benjamin–Hochberg procedure.

**SI Figure 1. Correlations between inflammatory markers.** The black lines demonstrate the three indexes (from left to right): LPS-induced inflammation index-1(composed of IL-10, IFN-γ, IL-2, IL-6, MMP-2, TNF-α, and TNF-β), LPS-induced inflammation index-2 (composed of IL-8, IL-18, MCP-1, MIP-1α, and MIP-1β), and basal inflammation index (composed of basal levels of CRP, IL-6, and TNF-α). Because LPS-induced markers were available for a subset of *n* = 1229 out of *n* = 2904 participants, the basal inflammation index was incomplete in the present figure.

**SI Figure 2. Associations of the basal inflammation index (*n* = 908), LPS-induced inflammation index-1 (*n* = 338), and LPS-induced inflammation index-2 (*n* = 364) with individual depressive symptoms during 9 years within a subsample of MDD patients.** Standardized beta coefficients with error bars representing standard errors of the predictive values of inflammatory indexes in relation to individual depressive symptoms during 9 years of follow up. Assessed using linear mixed models with repeated measures, adjusted for gender, age, use of anti-inflammatory drugs, and sickness prior to interview.

**SI Figure 3. Associations of the LPS-induced inflammation index with individual depressive symptoms during 9 years for the whole sample (*n* = 1147).** Standardized beta coefficients with error bars representing standard errors of the predictive values of inflammatory indexes in relation to individual depressive symptoms during 9 years follow-up. Assessed using linear mixed models with repeated measures adjusted for gender, age, use of anti-inflammatory drugs, and sickness prior to interview.

**SI Figure 4. Associations of the basal inflammation index (*n* = 2872), LPS-induced inflammation index-1 (*n* = 1147), and LPS-induced inflammation index-2 (*n* = 1229) with individual depressive symptoms during 9 years.** Standardized beta coefficients with error bars representing standard errors of the predictive values of inflammatory indexes in relation to individual depressive symptoms during 9 years of follow up. Assessed using linear mixed models with repeated measures, adjusted for chronic somatic diseases, antidepressants, gender, age, use of anti-inflammatory drugs, and sickness prior to interview.
