# Supplementary figures and images for: Basal and LPS-stimulated inflammatory markers and the course of individual symptoms of depression
Source: Transl Psychiatry. 2020 Jul 15;10:235. doi: 10.1038/s41398-020-00920-4 (PMC7363825; doi:10.1038/s41398-020-00920-4)

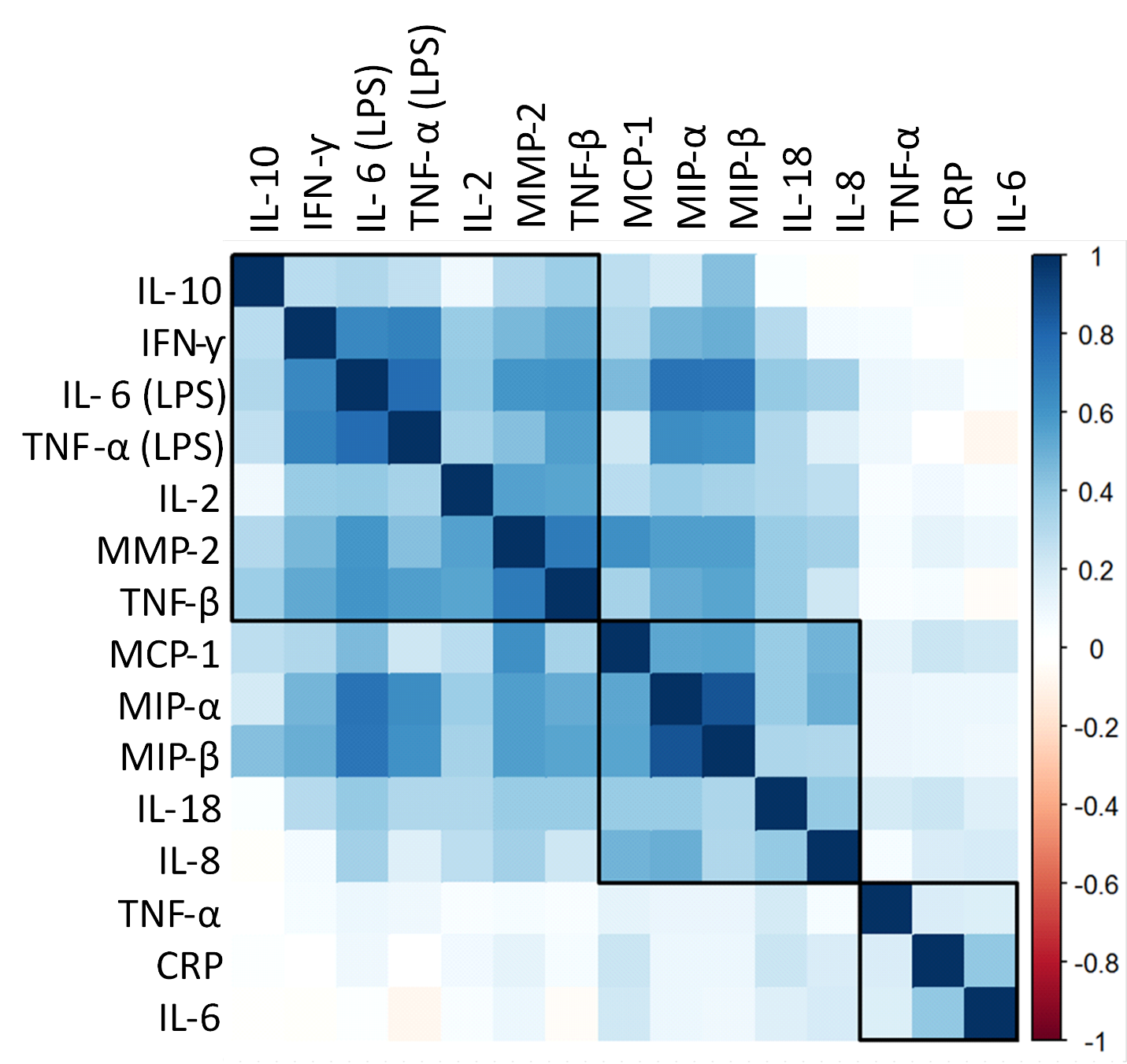

Supplement: Supplementary file 2 — Supplementary Figure 1 [file 41398_2020_920_MOESM2_ESM.tif]

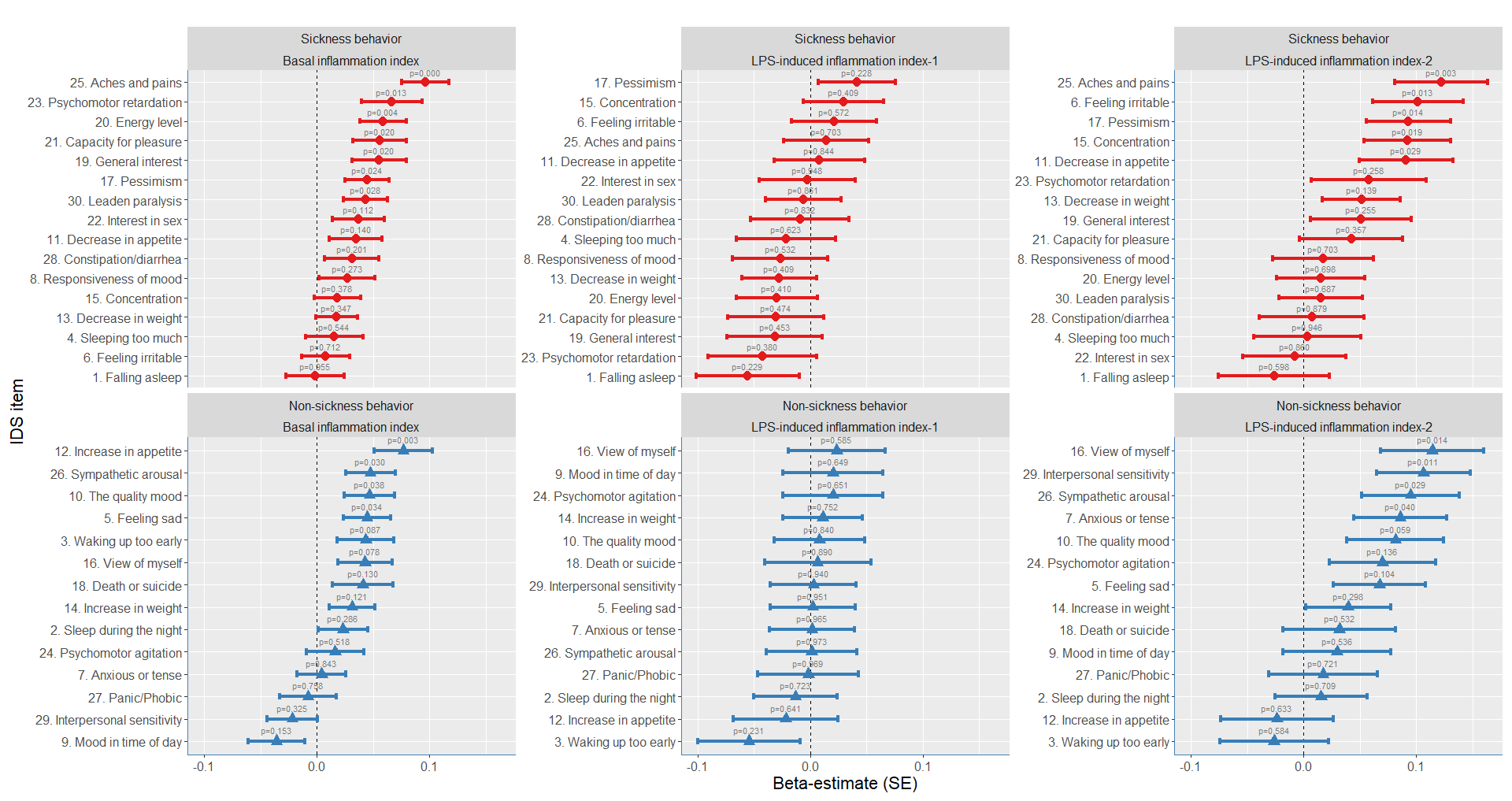

Supplement: Supplementary file 3 — Supplementary Figure 2 [file 41398_2020_920_MOESM3_ESM.tif]

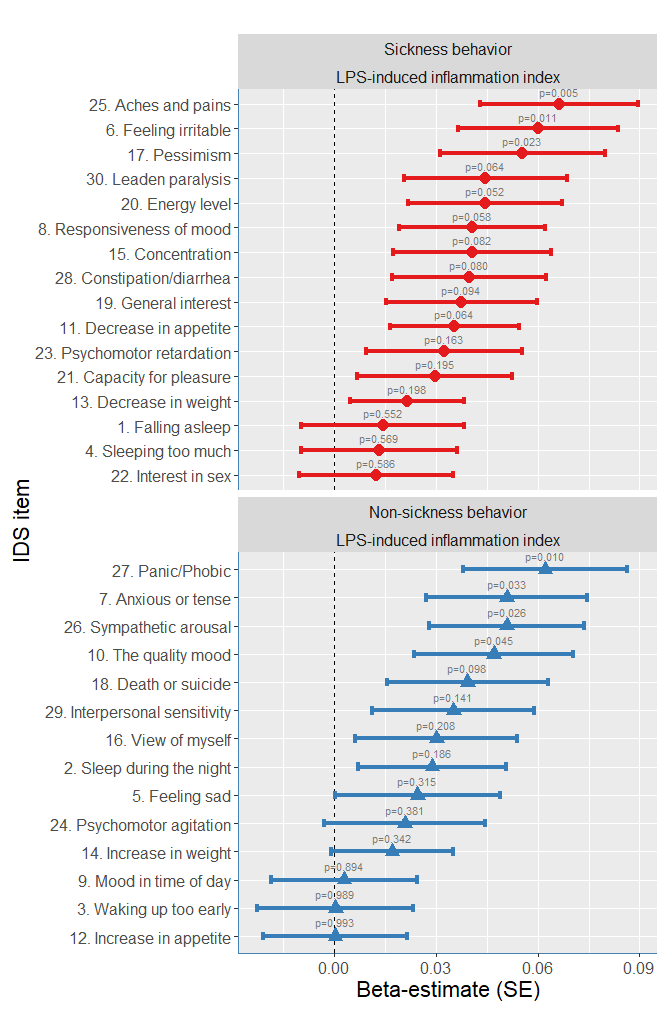

Supplement: Supplementary file 4 — Supplementary Figure 3 [file 41398_2020_920_MOESM4_ESM.tif]

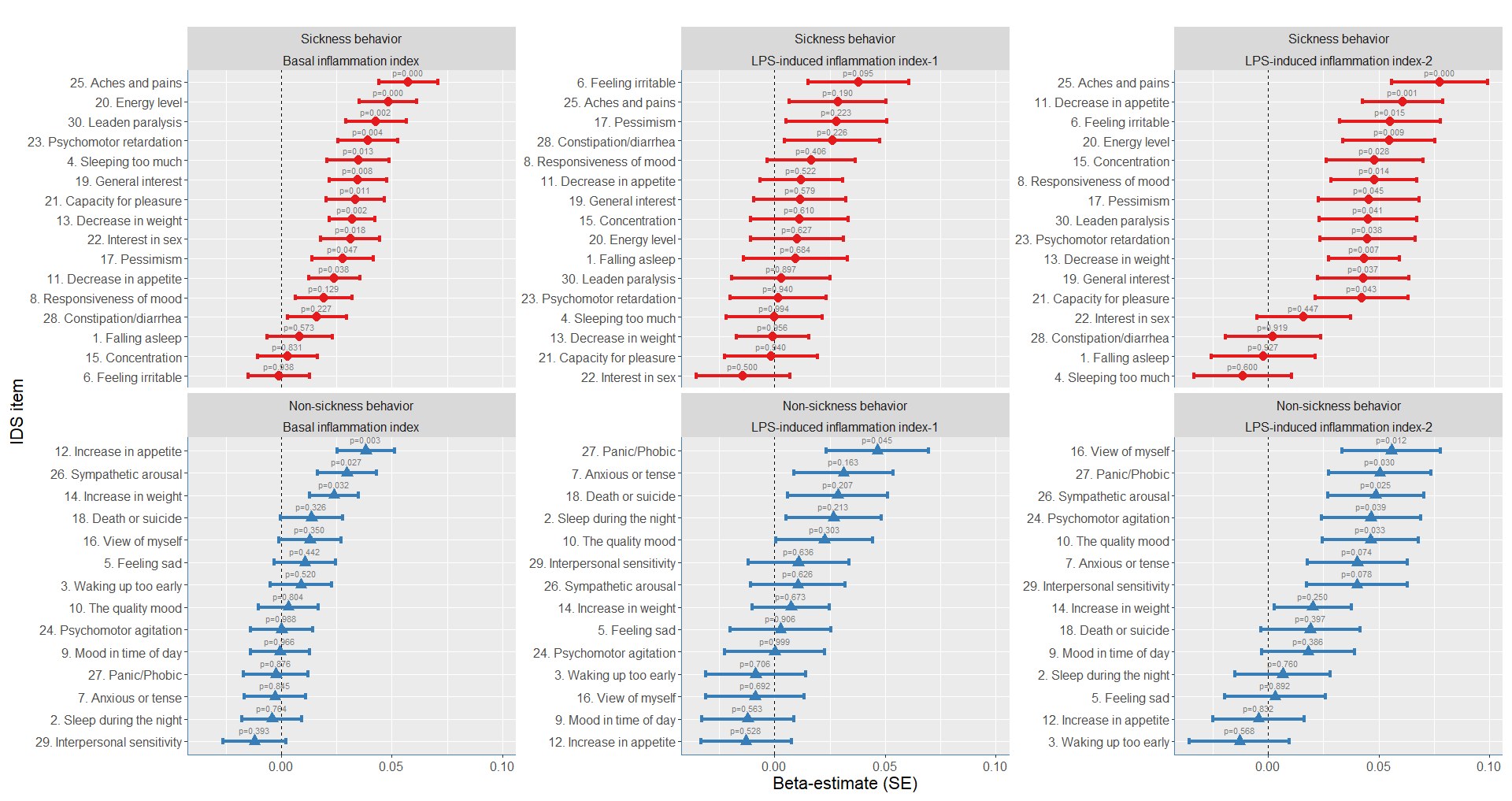

Supplement: Supplementary file 5 — Supplementary Figure 4 [file 41398_2020_920_MOESM5_ESM.tif]
